# A Zeb2-miR-200c loop controls midbrain dopaminergic neuron neurogenesis and migration

**DOI:** 10.1038/s42003-018-0080-0

**Published:** 2018-06-25

**Authors:** Shanzheng Yang, Enrique M. Toledo, Pedro Rosmaninho, Changgeng Peng, Per Uhlén, Diogo S. Castro, Ernest Arenas

**Affiliations:** 1 Laboratory for Molecular Neurobiology, Department of Medical Biochemistry and Biophysics, Karolinska Institutet, Solnavägen 9, 17177 Stockholm, Sweden; 2 Instituto Gulbenkian de Ciência, Rua da Quinta Grande, 6, 2780-156 Oeiras, Portugal

## Abstract

Zeb2 is a homeodomain transcription factor that plays pleiotropic functions during embryogenesis, but its role for midbrain dopaminergic (mDA) neuron development is unknown. Here we report that *Zeb2* is highly expressed in progenitor cells in the ventricular zone of the midbrain floor plate and downregulated in postmitotic neuroblasts. Functional experiments show that *Zeb2* expression in the embryonic ventral midbrain is dynamically regulated by a negative feedback loop that involves *miR-200c*. We also find that *Zeb2* overexpression reduces the levels of CXCR4, NR4A2, and PITX3 in the developing ventral midbrain in vivo, resulting in migration and mDA differentiation defects. This phenotype was recapitulated by *miR-200c* knockdown, suggesting that the Zeb2-miR-200c loop prevents the premature differentiation of mDA progenitors into postmitotic cells and their migration. Together, our study establishes Zeb2 and *miR-200c* as critical regulators that maintain the balance between mDA progenitor proliferation and neurogenesis.

## Abstract (web summary)

Shanzheng Yang et al. show that Zeb2 expression in the embryonic ventral midbrain is regulated by a negative feedback loop that involves miR-200c. This Zeb2-miR-200c loop provides novel insight into the mechanisms that properly cue the differentiation of midbrain dopaminergic neuronal progenitors.

## Introduction

The Zeb family of two-handed zinc finger/homeodomain proteins is formed by Zeb1 (also known as dEF1 or Zfhx1a) and Zeb2 (also known as Sip1 or Zfhx1b)^1^. De novo mutations in one allele of *ZEB2* gene cause the Mowat–Wilson syndrome, which is characterized by mental retardation, facial gestalt, and microcephaly^2^. In agreement with this, Zeb2 controls multiple aspects of the development of the nervous system, such as neural tube formation, neural crest specification, neural crest cell delamination, cerebral development, glial development, and spinal cord development^1^. A double-negative feedback loop involving Zeb1/2 and microRNAs of the *miR-200* family (*miR-200a, miR-200b, miR-200c, miR-141*, and *miR-429*) have been postulated to control epithelial–mesenchymal transition (EMT) in cancer cells^3–5^. In the forebrain, *Zeb1* mRNA was found strongly expressed in progenitor cells of the ventricular zone (VZ) around the lateral ventricles at E14 and E16 in the rat^6^. Zeb2 is also expressed during neurogenesis and differentiation of cortical and striatal interneurons^7,8^. Indeed, microRNAs of the *miR-200* family regulate the generation of forebrain neurons by targeting Zeb2^9^. In the ventral midbrain/hindbrain region, microRNAs of the *miR-200* family have been found to target the transcription factor SOX2 and the cell cycle regulator E2F3 in neural stem/progenitor cells^10^. A recent report showed that Zeb2 is expressed during dopaminergic neurogenesis, peaking on E12, and regulates axon growth and target innervation of dopaminergic neurons^11^. However, it is not known whether Zeb2 or *miR-200c* control midbrain dopaminergic (mDA) neurogenesis.

Understanding how mDA neurons are generated and maintained has become a particularly intense area of research because these cells degenerate in Parkinson’s disease (PD)^12^ and such knowledge may be relevant for the development of novel therapeutic strategies for PD. In the developing midbrain floor plate, neuroepithelial cells lining the ventricle give rise to all the cells in the entire mDA lineage. The interaction between at least two morphogen-regulated transcriptional networks, the Shh-Foxa2 and the Wnt1-Lmx1a-Lmx1b networks, controls the specification of dopaminergic progenitors in the VZ^13,14^. As development proceeds, dopaminergic progenitors exit cell cycle to undergo neurogenesis, a process that is controlled by proneural basic helix-loop-helix transcription factors, such as Neurog2 and Ascl1^15^, and nuclear receptors Nr1h3 and Nr1h2 (see refs.^16,17^). The proper control of this process is crucial, as precocious neurogenesis usually causes early depletion of the neural stem cell /progenitor pool^18^, resulting in decreased numbers of neurons. The first postmitotic cell generated in the dopaminergic lineage is the medial neuroblast^19^, a cell type characterized by the expression of the nuclear receptor *Nr4a2*, which is required for the generation of mDA neurons^20^. Medial neuroblasts express the chemokine receptor *Cxcr4* as they emerge in the intermediate zone (IZ) and initiate their radial migration toward the marginal zone (MZ) under the control of CXCL12, a chemokine, secreted by meninges^21^. While migrating, medial neuroblasts differentiate into a dopaminergic neuroblasts, characterized by the expression of *Pbx1*, a transcription factor required for the late specification, differentiation and survival of mDA neurons^22^. In its turn, PBX1 directly regulates the expression of *Pitx3* (see ref.^22^), a gene responsible for the differentiation and survival of mDA neurons^23–25^. In this study, we investigated the role of ZEB2 and *miR-200* in mDA neuron development. We found that *Zeb2* is expressed in VZ progenitors and downregulated in postmitotic neuroblasts by a negative feedback loop established with *miR-200c*. *Zeb2* gain-of-function or *miR-200c* knockdown reduced the levels of NR4A2 in the developing ventral midbrain, preventing the premature differentiation of mDA progenitors into postmitotic cells as well as their migration and maturation. Our results thus identify Zeb2 and *miR-200c* as important regulators of the balance between mDA progenitor maintenance and neurogenesis.

## Results

### *Zeb2* expression in the ventral midbrain

**Fig. 1:**
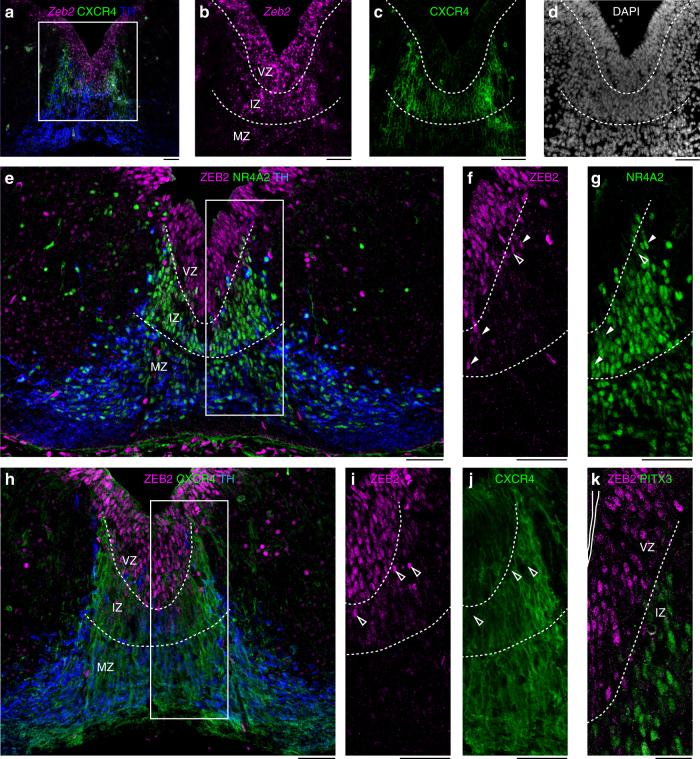
Zeb2 is expressed in developing midbrain. **a** In situ hybridization of *Zeb2* combined with immunofluorescence staining of CXCR4 (green) and tyrosine hydroxylase (TH; blue) reveal the expression of *Zeb2* in ventricular zone (VZ) and intermediate zone (IZ) in midbrain at E12.5. **b**, **c**, **d** Higher magnification images of boxed area in (**a**). *Zeb2* mRNA is localized in VZ and IZ. **c** CXCR4 is localized in IZ and marginal zone (MZ). **d** Section was counterstained with DAPI. **e** Immunofluorescence shows that ZEB2 (magenta) protein is localized in VZ. **f**, **g** Higher magnification images of boxed area in (**e**) show ZEB2 (**f**; magenta) and NR4A2 (**g**; green). White arrowheads in **f** and **g** show ZEB2^+^/NR4A2^+^ cells, whereas open arrowheads show ZEB2^+^/NR4A2^−^ cells in IZ. **h** Immunofluorescence shows that ZEB2 (magenta) is not colocalized with CXCR4 (green). **i**, **j** Higher magnification images of boxed area in (**h**) show ZEB2 (**i**; magenta) and CXCR4 (**j**; green). **k** Immunofluorescence shows that ZEB2 (magenta) is not colocalized with PITX3 (green). Open arrowheads in (**i**, **j**) show ZEB2^+^/CXCR4^−^ cells in IZ. Scale bars in all panel: 50 μm. IZ, intermediate zone; MZ, marginal zone; VZ, ventricular zone.

We first examined the expression of Zeb family members in the developing ventral midbrain. In situ hybridization (ISH) identified the expression of *Zeb2* in the ventricular zone (VZ) of the ventral midbrain, in the floor and basal plates, as well as in the intermediate zone (IZ) of the floor plate, together with CXCR4 at E12.5 (Fig. 1a-d). *Zeb1* was also detected in the midbrain floor plate, following a similar expression pattern, but at a lower level (Supplementary Figures 1 and 2). Immunofluorescence confirmed the presence of ZEB2 in SOX2^+^ progenitors of the VZ (Supplementary Figure 3). ZEB2 protein was also identified in a few NR4A2^+^/TH^−^ postmitotic dopaminergic neuroblasts of the IZ, near the VZ. However, the levels of ZEB2 immunoreactivity (IR) in such cells were lower compared to the VZ (Fig. 1e-g), suggesting a downregulation of Zeb2 as cells in the dopaminergic lineage differentiate. Indeed, ZEB2 and CXCR4 or PITX3 immuno reactivity in cells of the IZ were complementary (Fig. 1h-k), confirming the inverse relation between the expression of differentiation markers and Zeb2.

### ZEB2 binds to promoter regions of *Neurog2* and *Nr4a2*

**Fig. 2:**
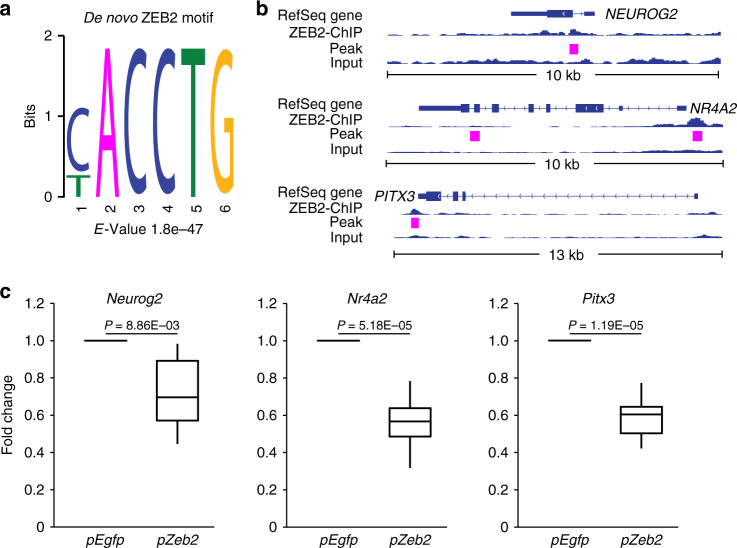
ZEB2 binds to promoters of *NR4A2*, *NEUROG2*, and *PITX3*. **a** ChIP-seq of ZEB2 identifies as de novo motif from ENCODE datasets (see Methods). **b** Genomic view of ZEB2 ChIP-seq reads and control input reads. In magenta regions identified as peaks by HOMER. **c** Quantitative RT-PCR shows that *Neurog2*, *Nr4a2*, and *Pitx3* mRNA were downregulated by *Zeb2* overexpression (*pZeb2*) in primary cells from embryonic ventral midbrain (E11.5) after 2 days (*n* = 6). Data were shown as mean ± SD. Student’s *t*-test was used.

In order to search for putative ZEB2 target genes with a role in ventral midbrain development, we analyzed chromatin immunoprecipitation-seqencing (ChIP-seq) datasets for ZEB2 made available by the ENCODE project^26^. A de novo search for sequences enriched at ZEB2 target regions identified the expected CACCTG motif (Fig. 2a) recognized by ZEB transcription factors, as previously described^27^. We found that ZEB2-binding events were located near the transcription start sites of *NEUROG2* and *NR4A2* genes (Fig. 2b), demonstrating the ability of ZEB2 to interact with the promoter of genes important for mDA neuron development. A binding event is also found immediately downstream of *PITX3* (Fig. 2b). In order to validate these results in dopaminergic cells, we overexpressed *Zeb2* in the substantia nigra dopaminergic cell line, SN4741 (see ref.^28^), and in primary ventral midbrain cells from embryonic day (E11.5). We found that *Zeb2* overexpression increased the levels of ZEB2 protein (Supplementary Figure 4A) and decreased the expression of *Neurog2* and *Pitx3* in dopaminergic SN4741 cells (Supplementary Figure 4B), and of *Neurog2, Nr4a2*, and *Pitx3* in primary embryonic midbrain cells (Fig. 2c). These observations suggested a possible role for Zeb2 in regulating diverse aspects of mDA neuron development such as neurogenesis and differentiation.

### *Zeb2* gain-of-function impairs mDA neurogenesis

**Fig. 3:**
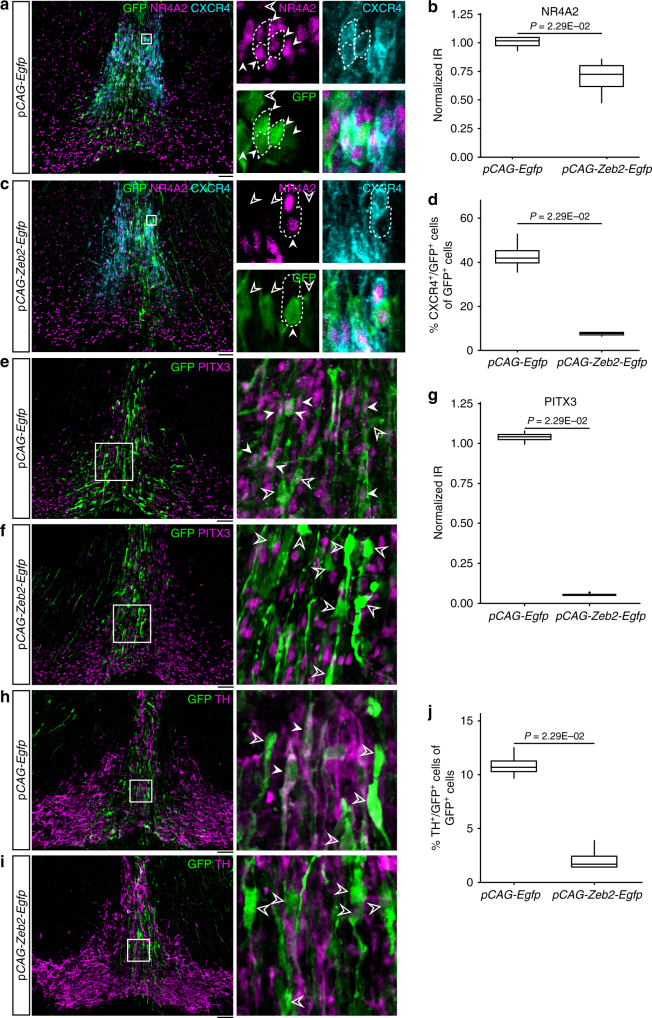
Overexpression of *Zeb2* reduces NR4A2 and PITX3 levels and impairs neurogenesis in the embryonic midbrain floor plate. **a**, **c** Mouse embryos were in utero electroporated with either *pCAG-Egfp* (**a**) control vector or *pCAG-Zeb2-Egfp* (**c**) to overexpress mouse *Zeb2* in the midbrain floor plate at E11.5. Boxed areas are magnified and each channel (NR4A2, magenta; CXCR4, turquoise; GFP, green) is shown. Dopaminergic linage was targeted by electroporation, as shown in (**a**, **c**), where full arrowheads indicate NR4A2^+^/GFP^+^ and open arrowheads indicate NR4A2^−^/GFP^+^ cells. **b** Quantification of NR4A2 immunoreactivity (IR) in GFP^+^ cells in *pCAG-Egfp* (*n* = 4; median 1.02, range 0.975–1.05) or *pCAG-Zeb2-Egfp* (*n* = 4; median 0.723, range 0.617–0.801) electroporated midbrain. **d** Quantification of the percentage of CXCR4^+^/GFP^+^ cells in total GFP^+^ cells in ventral midbrain dopaminergic domain in *pCAG-Egfp* (*n* = 4; median 41.9%, range 39.7–45.2%) or *pCAG-Zeb2-Egfp* (*n* = 4; median 7.71%, range 7.05–8.16%) electroporated midbrain. **e**, **f** Immunofluorescence images show the immunoreactivity of PITX3 (magenta) in GFP^+^ cells electroporated with *pCAG-Egfp* (**e**) or *pCAG-Zeb2-Gfp* (**f**). Boxed areas in **e** and **f** are magnified. White arrowheads indicate PITX3^+^/GFP^+^, whereas open arrowheads indicate PITX^−^/GFP^+^ cells. **g** Quantification of PITX3 IR in *pCAG-Egfp* (*n* = 4; median 1.04, range 1.02–1.06) or *pCAG-Zeb2-Egfp* (*n* = 4; median 0.0485, range 0.0453–0.0544) electroporated midbrain. **h**, **j** Immunofluorescence images show TH^+^/GFP^+^ cells in *pCAG-Egfp* (*n* = 4) or *pCAG-Zeb2-Egfp* (*n* = 4) electroporated midbrain. Boxed areas in **h** and **i** are magnified. White arrowheads indicate TH^+^/GFP^+^ while open arrowheads indicate TH^−^/GFP^+^ cells. **j** Quantification of the percentage of TH^+^/GFP^+^ cells in total GFP^+^ cells in ventral midbrain dopaminergic domain in *pCAG-Egfp* (*n* = 4; median 10.7%, range 10.3–11.3%) or *pCAG-Zeb2-Egfp* (*n* = 4; median 1.69%, range 1.41–2.44%) electroporated midbrain. Scale bars: 50 μm. Mann–Whitney *U*-test was used.

To examine the function of Zeb2 in mDA neurogenesis, we performed in utero electroporation of the ventral midbrain at E11.5 after intracerebroventricular injection of bi-cistronic plasmids encoding *Egfp* alone or mouse *Zeb2* and *Egfp*. Analysis of two postmitotic markers in the mDA lineage at E13.5 revealed a decrease in NR4A2 and PITX3 immunoreactivities in *Zeb2* overexpressing cells compared with control GFP^+^ cells (Fig. 3a-d, e-g). In agreement with the loss of these two factors required for the development of mDA neurons, we found that *Zeb2* overexpression reduced the percentage of TH^+^ mDA neurons from 10.7 to 1.69% (Fig. 3h-j), indicative of a role of ZEB2 in controlling mDA neurogenesis. This result was further validated by analysis of CXCR4, a marker present in NR4A2^+^ postmitotic neuroblast and neurons^21^. We found that *Zeb2* reduced the percentage of CXCR4^+^/GFP^+^ from 41.9 to 7.71% (Fig. 3a, c, d). Thus, our results show that Zeb2 inhibits mDA neurogenesis.

### *Zeb2* gain-of-function decreases mDA neuron migration

**Fig. 4:**
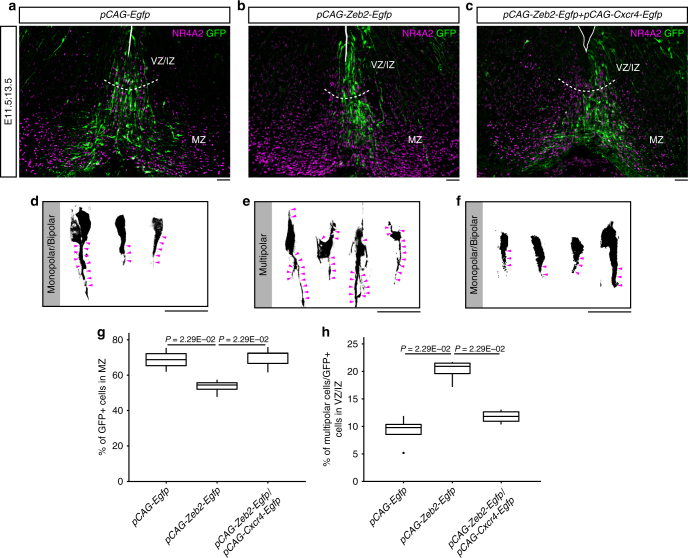
Overexpression of *Zeb2* alters radial distribution and cell morphology in embryonic midbrain. **a**–**c** Immunofluorescence images show the distribution of GFP^+^ cells in midbrain floor plate at E13.5. Embryos were electroporated at E11.5, with *pCAG-Egfp* **a**, *pCAG-Zeb2-Egfp* **b**, or *pCAG-Zeb2-Egfp* + *pCAG-Cxcr4-Egfp* (**c**). **d**–**f** GFP^+^ cells overexpressing *Zeb2* (**e**) show more multipolar morphology compared with monopolar/bipolar morphology in control GFP^+^ cells (**d**), whereas GFP^+^ cells co-electroporated with *pCAG*-*Zeb2-Egfp* + *pCAG-Cxcr4-Egfp* plasmids exhibit monopolar/bipolar morphology (**f**). **g** Percentage of GFP^+^ cells localized in MZ out of total GFP^+^ cells in midbrain floor plate of *pCAG-Egfp* (*n* = 4; median 68.9%, range 65.4–72.2%), *pCAG-Zeb2-Egfp* (*n* = 4; median 54.4%, range 52.1–55.8%) or *pCAG-Zeb2-Egfp* + *pCAG-Cxcr4-Egfp* (*n* = 4; median 69.5%, range 65.4–73.2%) electroporated midbrain. **h** Percentage of GFP^+^ cells displaying multipolar morphology in total GFP^+^ cells in VZ/IZ of *pCAG-Egfp* (*n* = 4; median 9.76%, range 8.55–10.4%), *pCAG-Zeb2-Egfp* (*n* = 4; median 20.9%, range 19.6–21.5%), or *pCAG-Zeb2-Egfp* *+* *pCAG-Cxcr4-Egfp* (*n* = 4; median 11.8%, range 10.9–12.6%) electroporated midbrain. Scale bars: 50 μm. Mann–Whitney *U*-test was used. IZ, intermediate zone; MZ, marginal zone; VZ, ventricular zone.

As Cxcr4 signaling mediates radial migration in mDA neurons^21,29^, we investigated the functional consequences of CXCR4 reduction by *Zeb2* overexpression at E11.5. Analysis of the efficiency of the *Egfp* and *Zeb2-Egfp* vectors revealed no significant difference in the proportion of GFP^+^ cells (Supplementary Figure 6). Electroporation of the control vector in the midbrain floor plate at E11.5 showed GFP^+^ cells reaching well into the marginal zone (MZ) at E13.5 and their processes changed orientation to start tangential migration. In contrast, in the *Zeb2* overexpression condition, fewer GFP^+^ cells entered the MZ and their processes did not change orientation (Fig. 4a, b and g), suggesting that *Zeb2* overexpression reduced radial migration. To confirm that this phenotype was due to a reduction in the levels of CXCR4, we performed a rescue experiment in which we simultaneously overexpressed *Zeb2* and *Cxcr4*. Indeed, *Cxcr4* gain-of-function largely rescued the number of GFP^+^ cells in the MZ and the orientation of their processes in the *Zeb2* condition (Fig. 4c, g and Supplementary Figure 5). Moreover, a detailed analysis of the morphology of the electroporated cells revealed that *Zeb2* overexpression increased the portion of multipolar cells from 9.76% in the control group to 20.9% (Fig. 4d, e and h), which was rescued to 11.8% by co-expression of *Cxcr4* (Fig. 4f and h). Notably these two alterations in *Zeb2* overexpressing cells phenocopied the migratory defects and the multipolar morphology that we previously reported in the *Cxcr4*^*-/-*^ mice or in wild-type mice treated with the CXCR4 antagonist, AMD3100 (see ref.^21^ and Supplementary Figure 7). Thus, combined, our results indicate that Zeb2 limits cell migration by reducing the levels of CXCR4 in vivo.

### *Zeb2* induces the expression of *miR-200c*

**Fig. 5:**
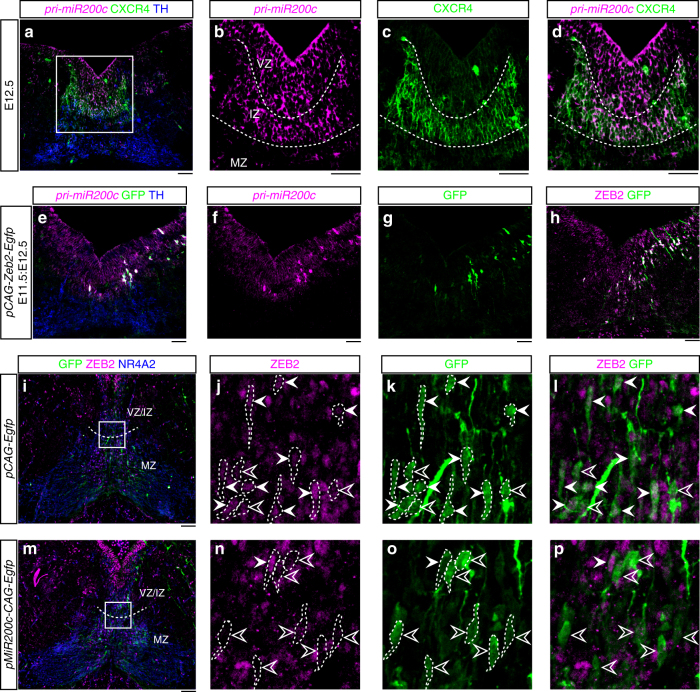
*MiR-200c* is expressed in midbrain floor plate and represses ZEB2. **a**, **b** In situ hybridization shows the expression of *primary miR-200c* (*pri-miR-200c*) in the VZ and IZ of the midbrain floor plate at E12.5. **b**–**d** Higher magnification images of boxed area in (**a**. **c**, **d**) In situ hybridization of *pri-miR-200c* and immunofluorescence staining of CXCR4 (green) show their colocalization in IZ. **e** *pCAG-Zeb2-Egfp* plasmids were electroporated into the midbrain floor plate at E11.5 and in situ hybridization was conducted to show *pri-miR-200c* at E12.5. Besides endogenous *pri-miR-200c* (magenta) in IZ of floor plate, high level of signal was also detected in GFP^+^ cells (green). **f**, **g** Individual channels show *pri-miR-200c* (**f**; magenta) and GFP (green). **h** Immunofluorescence image show the overexpression of ZEB2 in GFP^+^ cells. **i**–**p** The midbrain floor plate was electroporated with control vector, *pCAG-Egfp* (**i**–**l**), or *pMiR-200c-CAG-Egfp* (**m**–**p**) at E11.5 and analyzed at E13.5. The effects of *miR-200c* overexpression on ZEB2 were examined in a field (boxed area in **i** and **m**), centered in the boundary between the VZ and the IZ (dashed line), at the midline level. Boxes in **i** and **m** are magnified in **j**–**l** and **n**–**p**, respectively. ZEB2 immunoreactivity is much lower in most of GFP^+^ (green) cells overexpressing *miR-200c* (**n**–**p**) compared with GFP^+^ cells in *pCAG-Egfp* electroporated floor plate (**j**–**l**). White arrowheads indicate ZEB^+^/GFP^+^ cells while open arrowheads indicate ZEB^−^/GFP^+^ ones in **j**–**l** and **n**–**p**. Scale bars: 50 μm. IZ, intermediate zone; MZ, marginal zone; VZ, ventricular zone.

Previous studies have indicated that Zeb2 represses the expression of microRNAs of the *miR-200* family in cancer cells^3^, and that *miR-200c* targets *Zeb* mRNAs in different cell types^30^. We thus decided to investigate whether *miR-200c* controls any of the functions of ZEB2 in the ventral midbrain. We first examined the expression of primary *miR-200c* (*pri-miR-200c*) by ISH. Consistent with previous findings^10^ we found that *miR-200c* is expressed in the VZ of the midbrain floor plate. However, we also found *pri-miR-200c* expression in the IZ of the floor plate, in CXCR4^+^ cells (Fig. 5a and d), suggesting a possible role of *miR-200c* in regulating cell migration. We next examined possible cross-regulatory interactions between *Zeb2* and *miR-200c* in the mDA neuron lineage. To our surprise, we found that overexpression of *Zeb2-Egfp* by in utero electroporation in the ventral midbrain at E11.5, unlike *Egfp* alone, increases the expression of *pri-miR-200c* at E12.5 (Fig. 5e and h). On the other hand, overexpression of *pMiR-200c-CAG-Egfp* at E11.5, unlike *Egfp* alone, dramatically decreases the levels of ZEB2 in electroporated cells at E13.5 (Fig. 5i-p). Thus, our results indicate that Zeb2 negatively controls its own expression by establishing a negative feedback loop with *miR-200c*.

### Zeb2-miR-200c control mDA migration and neurogenesis

**Fig. 6:**
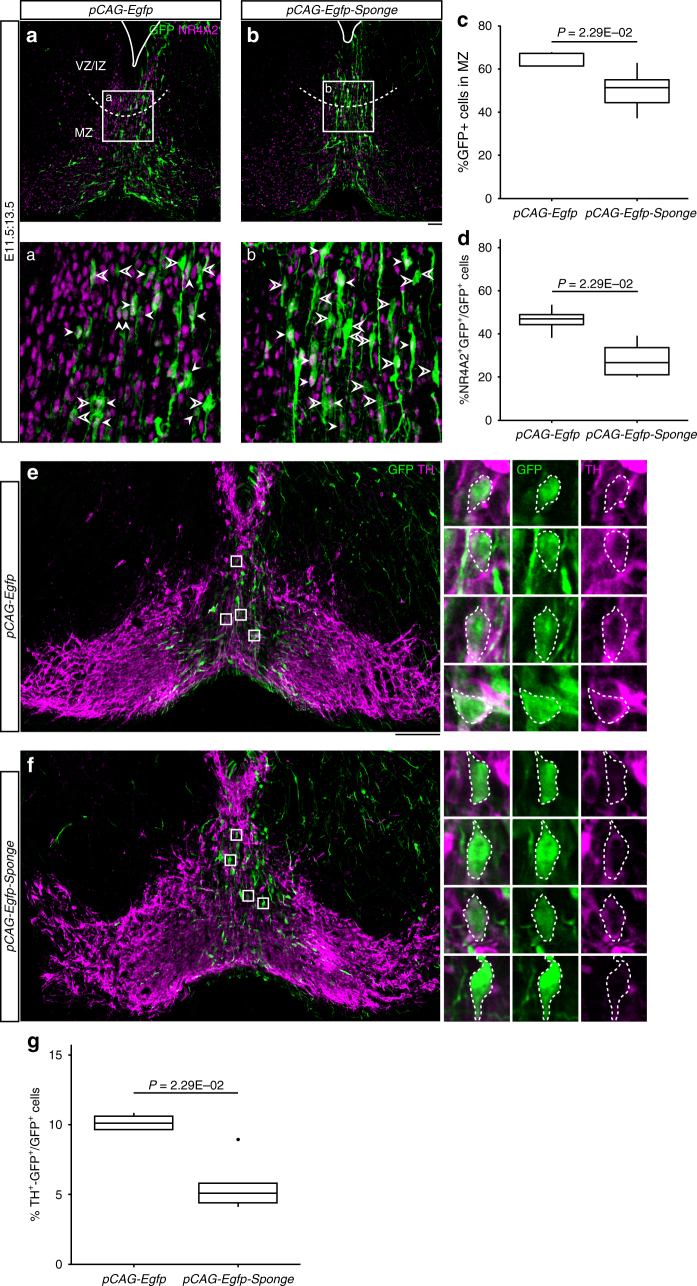
Knockdown of *miR-200*s impairs dopaminergic neurogenesis and radial migration in midbrain floor plate. **a**, **b** Immunofluorescence images show the distribution of GFP^+^ (green) cells at E13.5, which were electroporated at E11.5, with *pCAG-Egfp* control vector (**a**) or miR-200s sponge vector (*pCAG-Egfp-Sponge*; **b**) which antagonizes *miR-200* family microRNAs. Boxes a, b in (**a**, **b**) are magnified in **a** and **b**, respectively, to show colocalization of GFP (green) and NR4A2 (magenta). White arrowheads indicated NR4A2^+^/GFP^+^ cells, while open arrowheads show NR4A2^-^/GFP^+^ ones in (**a**, **b**. **c**) Percentage of GFP^+^ cells localized in MZ out of total GFP^+^ cells in floor plate of *pCAG-Egfp* (*n* = 5; median 61.4%, range 61.4–67.2%) or *pCAG-Egfp-Sponge* (*n* = 5; median 51.4%, range 44.4–55.0%) electroporated midbrain. **d** Percentage of NR4A2^+^/GFP^+^ cells out of GFP^+^ cells in floor plate of *pCAG-Egfp* (*n* = 5; median 47.0%, range 44.3–49.0%) or *pCAG-Egfp-Sponge* (*n* = 5; median 26.7%, range 21.1–33.7%) electroporated midbrain. **e**, **f** Immunofluorescence of TH (magenta) and GFP (green) in ventral midbrains electroporated with *pCAG-Egfp* control vector (**e**) or miR-200s sponge vector (*pCAG-Egfp-Sponge*; **f**). Boxes in **e** and **f** are magnified to show the colocalization of GFP (green) and TH (magenta). **g** Percentage of GFP^+^/TH^+^ cells in total GFP^+^ cells in floor plate of *pCAG-Egfp* (*n* = 5; median 10.1%, range 9.67–10.6%) or *pCAG-Egfp-Sponge* (*n* = 5; median 5.12%, range 4.41–5.81%) electroporated midbrain. Mann–Whitney *U*-test was used. Scale bar: 50 μm (**b** for **a** and **b**; **e** for **e** and **f**).

In order to further examine the function of the Zeb2-miR-200c negative feedback loop in mDA precursors, we knocked down *miR-200c* expression using a *miR-200 sponge* vector (*pCAG-Egfp-Sponge*) containing eight repeats of a fully complementary sequence to all five miR-200 family members inserted downstream of *Egfp* and driven by the CAG promoter^10^. First, we electroporated primary midbrain cells from E11.5 mouse embryos with *pCAG-Egfp*, *pCAG-Egfp-Sponge*, or *pMiR-200c-CAG-Egfp* and maintained the cells for 3 days in vitro. In agreement with the existence of a negative feedback loop, ZEB2 was elevated in *miR-200 Sponge*-overexpressing cells and downregulated in *miR-200c*-overexpressing cells (Supplementary Figure 8). Next, the sponge vector was electroporated in the midbrain floor plate of mouse embryos at E11.5 and brains were analyzed at E13.5. We found that *miR-200c* knockdown by the *miR-200 sponge* vector reduced the migration of GFP^+^ cells resulting in a decreased number of neurons reaching the MZ, compared with control *Egfp* (Fig. 6a-c). Moreover, as *Zeb2* overexpression reduced the levels of NR4A2 (Fig. 3c, d), we examined whether *miR-200c* knockdown also reduced the number of postmitotic NR4A2^+^ cells in the dopaminergic lineage and found that this was indeed the case (Fig. 6a,b and d). In line with this and previous observations, we also found that *miR-200c* knockdown reduced the number of mature TH^+^ dopaminergic cells (5.12% of total GFP^+^ cells), compared with the *Egfp* control (10.1% of total GFP^+^ cells) (Fig. 6e-g). Thus, overall, our results indicate that the negative Zeb2-miR-200c feedback loop controls not only migration but also mDA neurogenesis in vivo.

## Discussion

Our results identify the transcription factor ZEB2 and the microRNA *miR-200c* as part of a regulatory loop controlling the expression and function of pivotal genes, including *Neurog2* and *Nr4a2*, in mDA development. We found that Zeb2 induces the expression of *miR-200c*, which in turn, represses *Zeb2*. We suggest that high levels of Zeb2 in the VZ of the midbrain floor plate, increase the expression of *miR-200c* in mDA progenitor cells. Consistent with the role of microRNAs in posttranscriptional regulation, *miR-200c* negatively regulates *Zeb2*, resulting in reduced levels of ZEB2 protein, which in turn de-represses neurogenesis, resulting in more postmitotic cells in the IZ. Indeed, our results indicate that the function of ZEB2 in dopaminergic progenitors is to repress the expression of genes controlling mDA neurogenesis (*Neurog2*) and neuroblast differentiation (*Nr4a2*), preventing thus premature differentiation. We thus suggest that the balance between progenitor maintenance and neurogenesis/differentiation critically depends on a switch in the levels of ZEB2 and its regulation by *miR-200c*. Moreover, we also found that this mechanism indirectly de-represses *Cxcr4* expression, thereby controlling the radial migration and morphology of mDA neuroblasts and neurons.

The *miR-200* family has been shown to target Zeb1/2 and inhibit EMT in development and cancer^30,31^. Conversely, Zeb2 has been found to repress *miR-200* expression to promote EMT^3,4^. Although our results confirm that *miR-200c* also downregulates ZEB2 in the midbrain floor plate, we were surprised to find that Zeb2 upregulates *miR-200c*. This result suggests a regulatory loop with a different dynamics compared with other cellular contexts, but consistent with the finding that both Zeb2 and *miR-200c* are clearly colocalized in the VZ of the midbrain. Given the expression patterns and regulation that we observe, it is possible that ZEB2 directly controls *miR-200c*. Indeed, although ZEB proteins are thought to act primarily as transcriptional repressors by recruiting co-repressors, such as CtBP^32,33^ or NuRD^34–36^, they can also induce gene activation by recruiting co-activators such as the Specificity protein (Sp1) or Yes-associated protein (YAP1)^37–39^. Finally, it remains to be determined whether *Zeb1*, which is expressed at very low levels in the VZ of midbrain (present study) and regulates miR-200 family members^3,40^, may crosstalk with the Zeb2-miR-200 loop.

In the nervous system, Zeb2 has been previously found to serve several important functions. Zeb2 is for instance upregulated in the prospective neural plate where it contributes to BMP-Smad signaling repression and is required for neural induction^41^. Accordingly, *Zeb2* induces the expression of neural specific genes, such as *Sox2* and *Ncam*^42^, and is required for the development of specific cell lineages such as the neural crest^43^. ZEB proteins have been also found to regulate different aspects of cell polarity important for neural tube closure in the midbrain–hindbrain region^44^ and differentiation of granule neurons in the cerebellum^45^. Notably, in a recent study, Hegarty et al.^11^ has shown that Zeb2 negatively regulates mDA neuron axonal growth and target innervation, pointing out to Zeb2 as a negative regulator of mDA differentiation. Our results in this study show that Zeb2 additionally inhibits mDA neurogenesis, neuroblast differentiation into mDA neurons, and radial migration of mDA neurons. Combined, all current evidence supports a role of Zeb2 in preventing multiple aspects of mDA neuron differentiation. These neural functions, together with the known capacity of Zeb2 to induce an EMT program, underscores the importance of Zeb2 as an essential regulator of a broad range of basic processes in different biological systems.

In summary, our work identifies the Zeb2-*miR-200c* loop as an important mechanism controlling specific aspects of midbrain development such as mDA neurogenesis, neuroblast differentiation, and mDA neuron migration.

## Methods

### Animals

Male and female wild-type CD-1 mice (25–35 g; Charles River) were housed, bred, and treated according to local ethical committees: Stockholm Norra Djurförsöksetisks Nämnd (N273/11, N370/09, N486/12, and N40/15).

For embryo analyses, wild-type CD-1 mice were mated overnight and noon of the day the plug was considered E0.5. Embryos were dissected out of the uterine horns in ice-cold phosphate-buffered saline (PBS), fixed in 4% (wt/wt) paraformaldehyde (PFA) for 4 h to overnight, cryoprotected in 15–30% sucrose, and frozen in Tissue-Tek Optimum Cutting Temperature (OCT) compound (Sakura Fine-Tek, Tokyo, Japan) on dry ice. Serial coronal 14 μm sections of the brain were obtained on a cryostat.

### Plasmid constructs

A mouse *Zeb2* cDNA was purchased from Dharmacon (catalog number: MMM1013-202798430; clone ID: 6408845). The coding sequence (CDS) of mouse *Zeb2* was amplified by PCR using the following primers: forward 5′-ataGAATTCatgaagcagccgatcatggc-3′ (EcoRI) and reverse 5′-cgCTCGAGttattccatgccatcttccatattg-3′ (XhoI). The CDS of mouse *Zeb2* was then cloned into the *pCAGIG* vector^21,46^ in between the EcoRI and XhoI restriction sites to obtain the *pCAG-Zeb2-IRES-Egfp* plasmid (referred to as *pCAG-Zeb2-Egfp*). The CDS of human *CXCR4* was sub-cloned from a *CXCR4-Gfp* plasmid, kindly provided by Dr. Stefano Marullo (Institut Cochin, France) and Dr. Hélène Boudin (Université de Nantes, France), into the *pCAGIG* vector in between NheI and NotI sites to obtain *pCAG-CXCR4-IRES-Egfp* (referred to as *pCAG-Cxcr4-Egfp*). The construction of *miR-200c* overexpression and sponge vectors were described previously^10^. Briefly, a U6 promoter driven *miR-200c* overexpression cassette (harboring *mmu-miR-200c* gene amplified from C57BL/6 mouse genomic DNA by PCR) was sub-cloned into *pCAG-IRES-Egfp* vector to obtain *pU6-miR-200c-CAG-IRES-Egfp* (referred to as *pMiR-200c-CAG-Egfp*). Oligonucleotides that recognize the five members of the *miR-200* family were synthesized, annealed, ligated, gel purified, and cloned as concatemers into the *pCAG–IRES-Egfp* vector to obtain *pCAG–Egfp–8* × *_miR-200 sponge* plasmid (referred to as *pCAG-Egfp-sponge*). In addition, we also verified that transfection of different constructs (*pCAG-Egfp, pCAG-Zeb2-Egfp* and *pMiR-200c-CAG-Egfp*), through a range of concentrations, give rise to comparable numbers of GFP^+^ cells in SN4741 cells (Supplementary Figure 6).

### Cell line, primary cultures, and electroporation

The mouse SN4741 dopaminergic-like cell line was cultured in Dulbecco’s modified Eagle’s medium (DMEM; Invitrogen) supplemented with 10% fetal bovine serum (FBS; Invitrogen), 1% penicillin–streptomycin (Invitrogen), and 1% l-glutamine (Invitrogen)^47^. For the purpose of electroporation, cells were trypsinized and suspended. Mouse ventral midbrain primary cells were obtained as previously described^48,49^. Briefly, ventral midbrain tissue was dissected from E11.5 mouse embryos, dissociated with collagenase/dispase (30 min at 37 °C on a rocking platform), followed by mechanical trituration. For electroporation, cell suspension were aliquoted and electroporated with different plasmids according to manufacturer’s manual (Ingenio^®^ from Mirus Bio, Madison, Wisconsin). The following constructs were used: *pCAG-IRES-Egfp*, *pCAG-Zeb2-Egfp*, *pCAG-Egfp-Sponge*, and *pMiR-200c-CAG-Egfp*. After electroporation, SN4741 cells were plated in warm medium (10% FBS in DMEM, supplemented with 1% l-glutamine). Primary cells were plated at a density of 100,000 cells cm^−2^ in N2 medium (Ham’s F-12 medium and minimal essential medium, supplemented with HEPES, N2 supplement, and l-glutamine, all from Invitrogen) in 48-well plate. Cells were subjected to fixation for immunocytochemistry or RNA extraction 48 h after electroporation.

### Immunohistochemical analysis

Sections were pre-incubated for 1 h in blocking solution followed by incubation at 4 °C overnight with following primary antibodies: chicken anti-GFP (1:1000; Aves Labs, Inc., Tigard, Oregon**)**, rabbit anti-NR4A2 (1:200; Santa Cruz Biotechnology, Inc., Dallas, Texas), mouse anti-NR4A2 (1:200; R&D Systems, Minneapolis, Minnesota), rabbit anti-TH (1:750; Pel-Freez Biologicals, Rogers, Arkansas), sheep anti-TH (1:500; Novus Biologicals, Littleton, Colorado), rabbit anti-CXCR4 (1:200; Epitomics, Burlingame, California), rabbit anti-ZEB2 (1:150; Novus Biologicals), and goat anti-SOX2 (1:200; Abcam, Cambridge, UK). After washing, slides were incubated for 1–2 h at room temperature with the appropriate fluorophore-conjugated (Alexa488-, 1:500, Jackson Laboratories, Bar Harbor, Maine; Alexa488-, 555-, and 647-, 1:1000, Thermo Fisher Scientific, Waltham, Massachusetts) secondary antibodies. Confocal pictures were taken with a Zeiss LSM700 microscope. Cell distribution and density were used to define the border between the VZ/IZ and the MZ.

For lambda protein phosphatase (λPP) treatment, sections were incubated with λPP (800U; New England Biolabs, Ipswich, Massachusetts) in Protein MetalloPhosphatases buffer with MnCl_2_ provided by manufacturer for 1 h at room temperature. Control sections were subjected to the same process, except in a buffer without λPP. After λPP treatment, sections were rinsed in PBS for three times and followed by regular immunohistochemistry process.

Fiji software^50,51^ was used to quantification of IR. The mean intensity of CXCR4 fluorescence of GFP^+^/NR4A2^+^ cells was normalized to the average of the mean intensity of two to three neighboring GFP^−^/NR4A2^+^ cells. For quantification of IR of NR4A2 or PITX3, the mean intensity of NR4A2 or PITX3 fluorescence of GFP^+^ cells was normalized to the average of the mean intensity of 2-3 neighboring GFP^−^ cells within dopaminergic domain in floor plate. The normalized CXCR4/NR4A2/PITX3 IR of GFP^+^ cells was averaged in one section. For quantification of IR of ZEB2 in electroporated primary ventral midbrain cells, the mean intensity of ZEB2 fluorescence of GFP^+^ cells was normalized to the average of the mean intensity of two to three neighboring GFP^−^ cells. Data present median and interquartile range of all cells in each group (*pCAG-Egfp*, *pCAG-Egfp-Sponge*, and *pMiR-200c-CAG-Egfp*) from three independent experiments (three litters for each experiment).

### In situ hybridization

For ISH, embryos were fixed (4% PFA, 4 °C) overnight before being cryopreserved in 30% sucrose, frozen in OCT and sectioned at 14 μm. The template for RNA probe recognizing *miR-200c* primary transcript was amplified from mouse genomic DNA. The templates for RNA probe synthesis (*Zeb1* and *Zeb2*) were amplified from cDNA of mouse SN4741 cells by PCR using primers listed in Table 1. DNA polymerase Phusion was used according to manufacturer’s instruction (New England Biolabs). PCR product was subjected to agarose electrophoresis and was purified using the QIAquick Gel Extraction Kit (Qiagen, Hilden, Germany). Transcription of digoxigenin (DIG)-labeled riboprobes was carried out using SP6 RNA Polymerase (Thermo Fisher Scientific) according to manufacturer’s instruction. ISH was performed on fixed tissue with DIG-labeled single-stranded RNA probes at 65 °C, followed by alkaline-phosphatase-coupled anti-DIG antibody recognition^52^ and incubation with Fast red substrates (Roche).

**Table 1:** Primers used for cloning of templates for riboprobes for in situ hybridization

Gene	Sense primer (T7 underlined)	Antisense primer (SP6 underlined)	Reference Sequence
*Primary miR-200c*	5′-gagTAATACGACTCACTATAGGAAGGAGGAAGAGCGAGAGTG-3′ (124718826–124718807)	5′-gagATTTAGGTGACACTATAGACATCATTACCCGGCAGTATTAGAG-3′ (124718326–124718349)	NC_000072.6
*mZeb1*	5′-gagTAATACGACTCACTATAGGCGTTGGCTCATTCTTAAGCTCT-3′ (3724–3745)	5′-gagATTTAGGTGACACTATAGATGCTTCAGCTTCTCTCACAGTC-3′ (4342–4321)	NM_011546.3
*mZeb2*	5′-gagTAATACGACTCACTATAGGTAAGGGAGAGTGTTGTGGAG-3′ (620–639)	5′-gagATTTAGGTGACACTATAGAGGGCTTCTGGGTAAATAATGG-3′ (895–875)	NM_001355289.1

Numbers in parentheses indicate the location of the primer binding site in the reference sequence

### Quantitative RT-PCR

Total RNA was isolated using RNeasy kit (Qiagen) and cDNA was made with cDNA SuperScript II Reverse Transcriptase kit (Thermo Fisher Scientific). Specific genes were amplified using Fast SYBR Green Master mix kit (Thermo Fisher Scientific). Real-Time PCR was performed using fast protocols on a 7500 Fast Real-Time PCR system (Thermo Fisher Scientific). *Gapdh* was used to normalize the expression of mRNA. Primers used are listed in Table 2.

**Table 2:** Primers for qRT-PCR

Gene	Forward primer	Reverse primer
Mouse *Gapdh*	5′-tggcctccaaggagtaagaa-3′	5′-tgtgagggagatgctcagtg-3′
Mouse *Neurog2*	5′-agctcctcgtcctcctcct-3′	5′-gacattcccggacacacac-3′
Mouse *Nr4a2*	5′-tcgacatttctgccttctcc-3′	5′-ccactctcttgggttccttg-3′
Mouse *Pitx3*	5′-ttcccgttcgccttcaactcg-3′	5′-gagctgggcggtgagaatacagg-3′

### In utero electroporation

E12.5 pregnant females were deeply anesthetized using Isofluorane (IsoFlo®, Abbott Labs, Lake Bluff, Illinois) and the uterine horns were accessed through an abdominal incision. Approximately 1 µl of *pCAG-Egfp*, *pCAG-Zeb2-Egfp*, *pCAG-Cxcr4*^*−*^*Egfp*, *or pCAG-Egfp-Sponge* plasmids were injected into the mesencephalic ventricle. Plasmids were used at 1 µg µl^−1^ in PBS containing 10% of Fast Green (Sigma). Square electric pulses of 30 V and 50 ms were passed through the uterus five times, spaced 950 ms, using a square pulse electroporator (ECM 830, Harvard Apparatus, Holliston, Massachusetts). The uterine horns were replaced into the abdominal cavity, which was then closed with sutures.

### Western blotting

For western blot analysis, SN4741 cells were washed twice with PBS (pH 7.2) and lysed in RIPA buffer (150 mM NaCl, 50 mM Tris·Cl pH 7.4, 1 mM EDTA, 0.5% NP-40, 1 × protease inhibitor cocktail [Roche]). Protein concentrations were determined using DC Protein Assay Kit (Bio-Rad, Hercules, CA). Equal amounts of total protein were subjected to 10% SDS-polyacrylamide gel electrophoresis, and electro-transferred onto polyvinyl difluoride membrane. After blocking with 5% bovine serum albumin, the membranes were incubated with goat anti-ZEB2 antibody (LifeSpan BioSciences, Inc., Seattle, WA) followed by a secondary antibody (anti-goat IgG, peroxidase conjugated, from Sigma-Aldrich, Saint Louis, MO). Bands were visualized by luminescence using the ECL prime reagent (Amersham, Aylesbury, UK) according to manufacturer’s instructions and the Chemi-Doc XRS system (Bio-Rad). After detection of ZEB2, membranes were stripped in 62.5 mM Tris/HCl (pH 6.8), 2% SDS, and 100 mM β-mercaptoethanol, washed, re-blotted with a mouse anti-GAPDH antibody, followed by an anti-mouse IgG peroxidase-conjugated antibody (Sigma-Aldrich), and detected by luminescence.

### Bioinformatics analysis

ChIP-seq alignment files from ENCODE project^26^ were retrieved from the database. Their Encode accession numbers are as follows: ZEB2 ChIP-seq (ENCSR322CFO), control input (ENCSR173USI), done in K562 cells.

Peak finding and gene annotation was done with HOMER v4.9 software^53^ for each cell type, with the following settings: -F = 3, -LP = 0.0001, -size = 200, -minDist = 500. ChIP-seq transcripts profiles were done with integrative genome viewer software^54^ v2.3.97. De novo motif finding for ZEB2 ChIP-seq were performed using the MEME-suit, MEME-ChIP^55^ v4.11.2 with default options.

### Statistical analyses

Statistical analyses (Student’s *t*-test and Mann–Whitney *U*-test) were performed using R^56^ v3.4.3. *P*-values of <0.05 were considered statistically significant differences. Results are presented as mean ± SD or median and interquartile range.

### Data availability

The ChIP-seq datasets analyzed during the current study were published by ENCODE project and have been deposited into GEO with the following accession codes GEO:GSE91663 (ZEB2; ENCSR322CFO) and GEO:GSE91548 (Control; ENCSR173USI). Other data are available from the corresponding author upon reasonable request.

## Electronic supplementary material

Supplementary information
