# Supplementary material for: A Zeb2-miR-200c loop controls midbrain dopaminergic neuron neurogenesis and migration
Source: Commun Biol. 2018 Jun 25;1:75. doi: 10.1038/s42003-018-0080-0 (PMC6123725; doi:10.1038/s42003-018-0080-0)
Supplement: Supplementary file 1 — Supplementary information [file 42003_2018_80_MOESM1_ESM.pdf]

Supplementary Figures

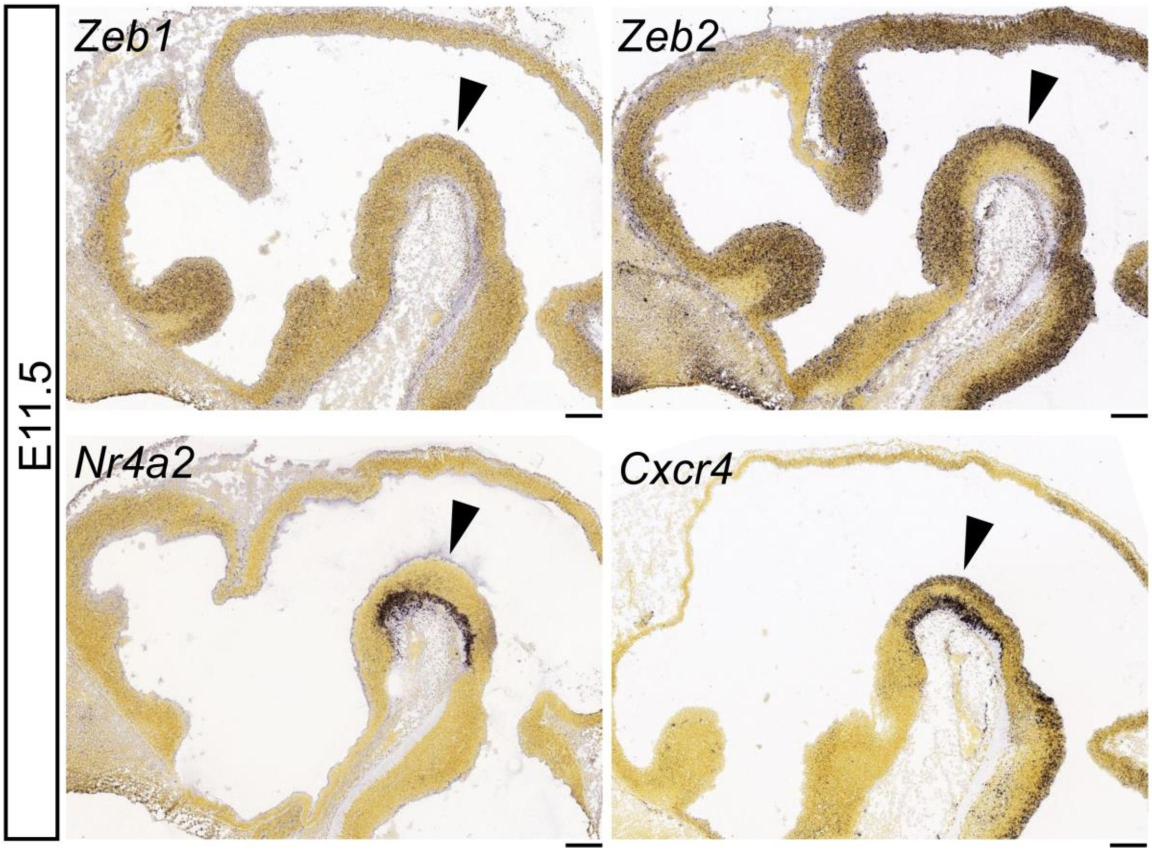

Supplementary Figure. 1. In situ hybridization of *Zeb1*, *Zeb2*, *Nr4a2* and *Cxcr4* (image data from Allen Institute for Brain Science (2008). Allen Developing Mouse Brain Atlas. Available from: http://developingmouse.brain-map.org). In situ hybridization done in mouse sagittal sections at E11.5 show that *Zeb1* (at a much lower level than *Zeb2*) and *Zeb2* is expressed in VZ. Arrowheads point to the ventricular zone of the midbrain floorplate. Scale bars: 200 μm.

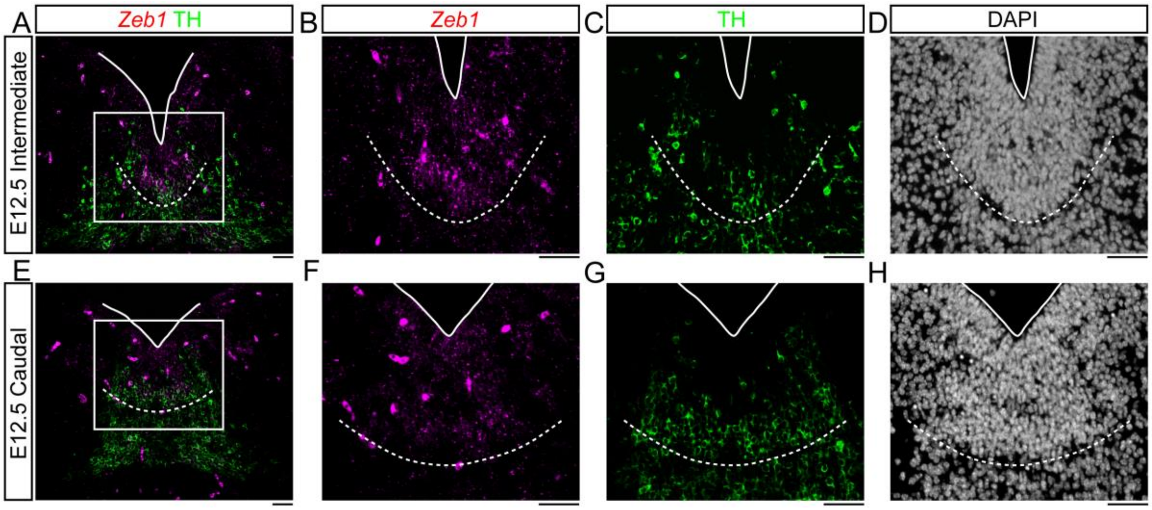

Supplementary Figure 2. In situ hybridization of *Zeb1* in midbrain floor plate at E12.5. Messenger RNA of *Zeb1* (magenta) is barely detected by in situ hybridization at intermediate (A-D) and caudal levels (E-H) of midbrain. Immunofluorescence staining of tyrosine hydroxylase (TH; green; A, C, E, G) marks mature dopaminergic neurons. DAPI staining (D, H) shows the nuclei in boxed areas in A and E. Scale bars: 50 μm.

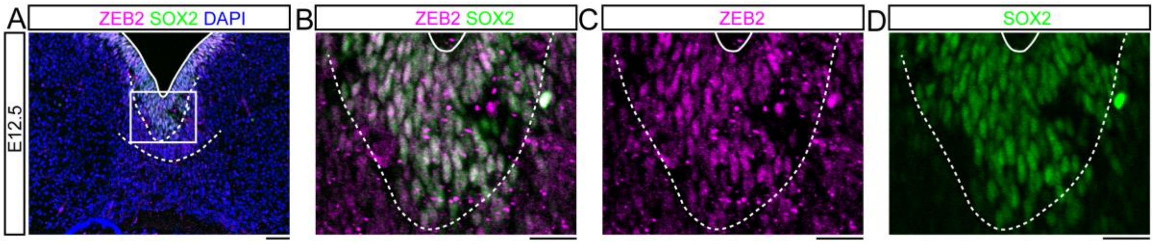

Supplementary Figure 3. ZEB2 is co-localized with SOX2 in the ventricular zone of the midbrain floor plate at E12.5. (A) Immunofluorescence image shows ZEB2 (magenta) and SOX2 (green) are localized in ventricular zone (VZ). (B-D) Higher magnification images of boxed area in (A). Scale bars: 50 μm (A); 20 μm (B-D).

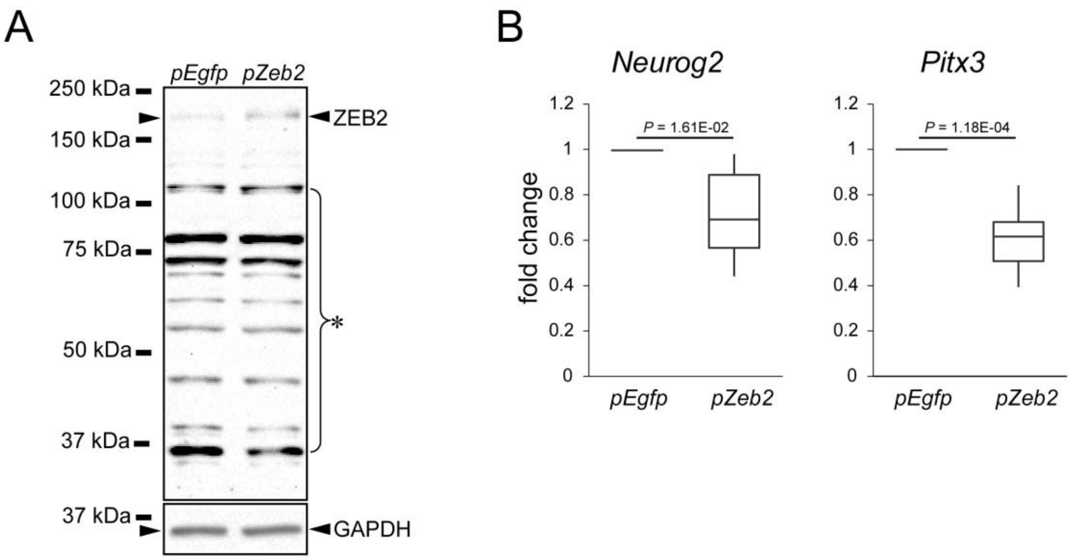

Supplementary Figure 4. Overexpression of *Zeb2* in SN4741 cells reduces the expression of *Neurog2* and *Pitx3*. (A) Western blot shows that overexpression of *pCAG-Zeb2-Egfp (pZeb2)* increased the levels of ZEB2 protein compared to control (*pCAG-Egfp*; *pEgfp*) in SN4741 cells two days after electroporation (n = 3). The molecular weight of ZEB2 band detected is 180 kDa, consistent with previous reports[1–3]. *Non-specific bands with lower molecular weight. (B). Q-RT-PCR shows that overexpression of *Zeb2* downregulated *Neurog2* and *Pitx3* in SN4741 cells two days after electroporation (n = 5). Data show mean ± SD. Student t-test was used was used for statistical analysis.

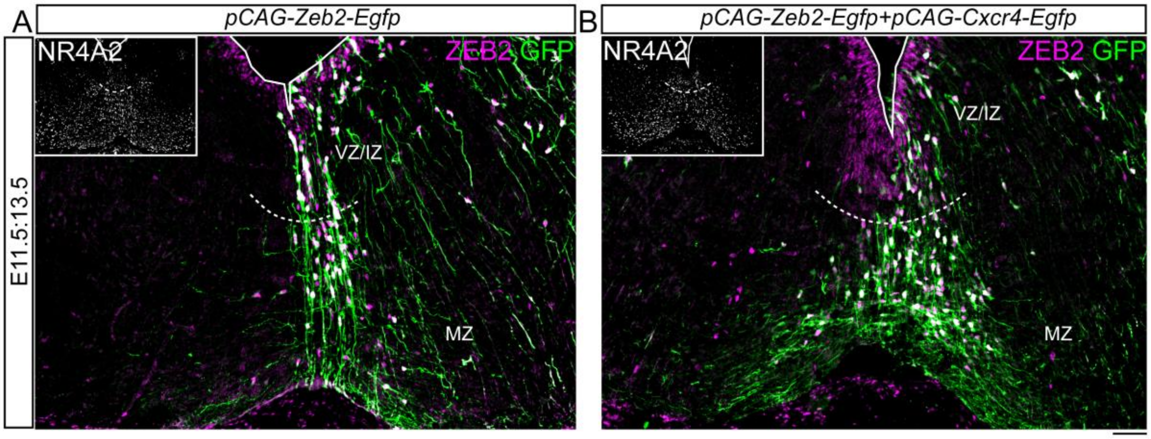

Supplementary Figure 5. *Cxcr4* rescues the migration deficits induced by *Zeb2* overexpression. The midbrain floor plate was electroporated with *pCAG-Zeb2-Egfp* (A) or *pCAG-Zeb2-Egfp+pCAG-Cxcr4-Egfp* (B) at E11.5. Analysis was performed by immunofluorescence at E13.5 to identify NR4A2$^+$ cells in the dopaminergic lineage (grey; insert) as well as ZEB2$^+$ cells (magenta) and electroporated cells (GFP$^+$, green). Scale bars: 50 μm.

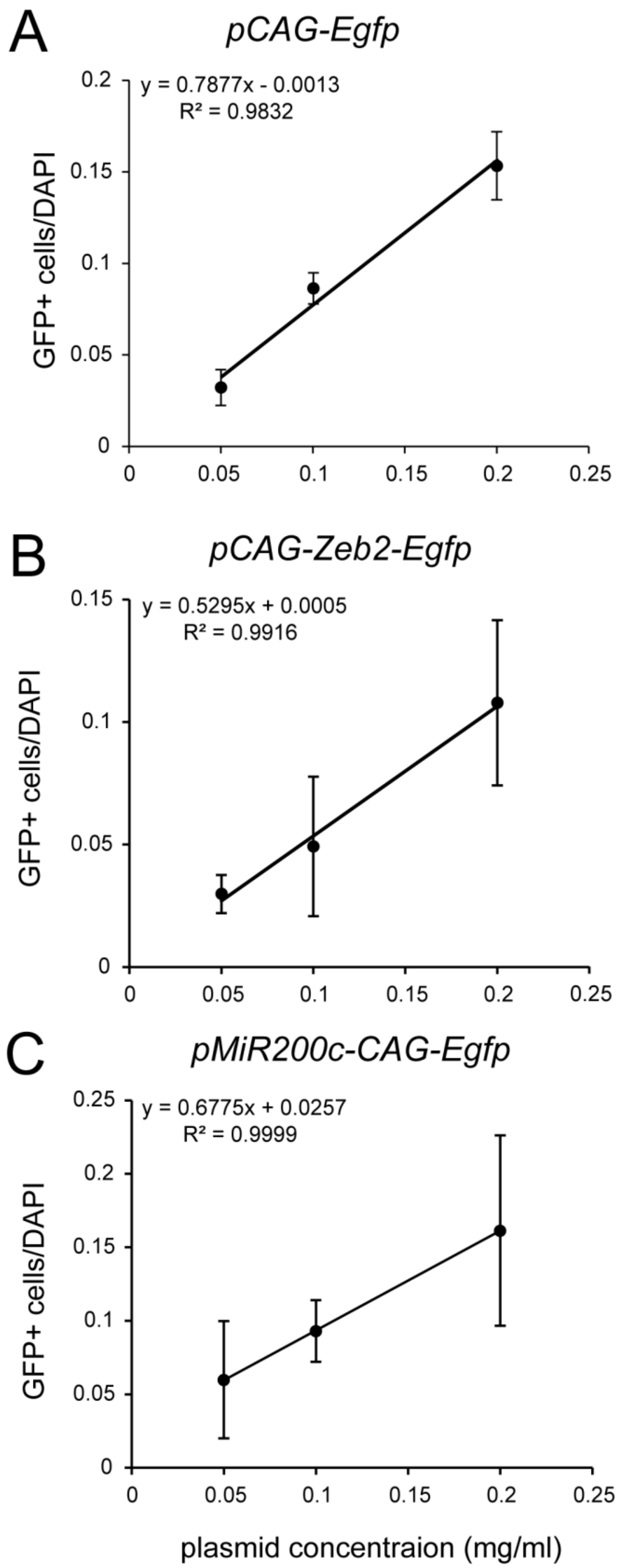
A
pCAG-Egfp
y = 0.7877x - 0.0013
R² = 0.9832
GFP+ cells/DAPI
0.2
0.15
0.1
0.05
0
0
0.05
0.1
0.15
0.2
0.25
B
pCAG-Zeb2-Egfp
y = 0.5295x + 0.0005
R² = 0.9916
GFP+ cells/DAPI
0.15
0.1
0.05
0
0
0.05
0.1
0.15
0.2
0.25
C
pMiR200c-CAG-Egfp
y = 0.6775x + 0.0257
R² = 0.9999
GFP+ cells/DAPI
0.25
0.2
0.15
0.1
0.05
0
0
0.05
0.1
0.15
0.2
0.25
plasmid concentraion (mg/ml)

Supplementary Figure 6. Dose response analysis of the proportion of GFP^+ cells after electroporation of different constructs used in this study. Different constructs with the same backbone and promoter (*pCAGIG)* but different inserts were tested. *pCAG-Egfp* (A); *pCAG-Zeb2-Egfp* (B); and *pMiR200c-CAG-Egfp* (C) were electroporated into SN4741 cells at different concentration in the same setting for electroporation (n = 3). The results show comparable efficiencies and a good correlation between plasmid concentration and efficiency, as shown by the coefficient of determination (R^2). No significant difference was detected between the efficiency of the *Egfp* and *Zeb2-Egfp* constructs at 0.2 mg/ml by Student t test ($P > 0.05$).

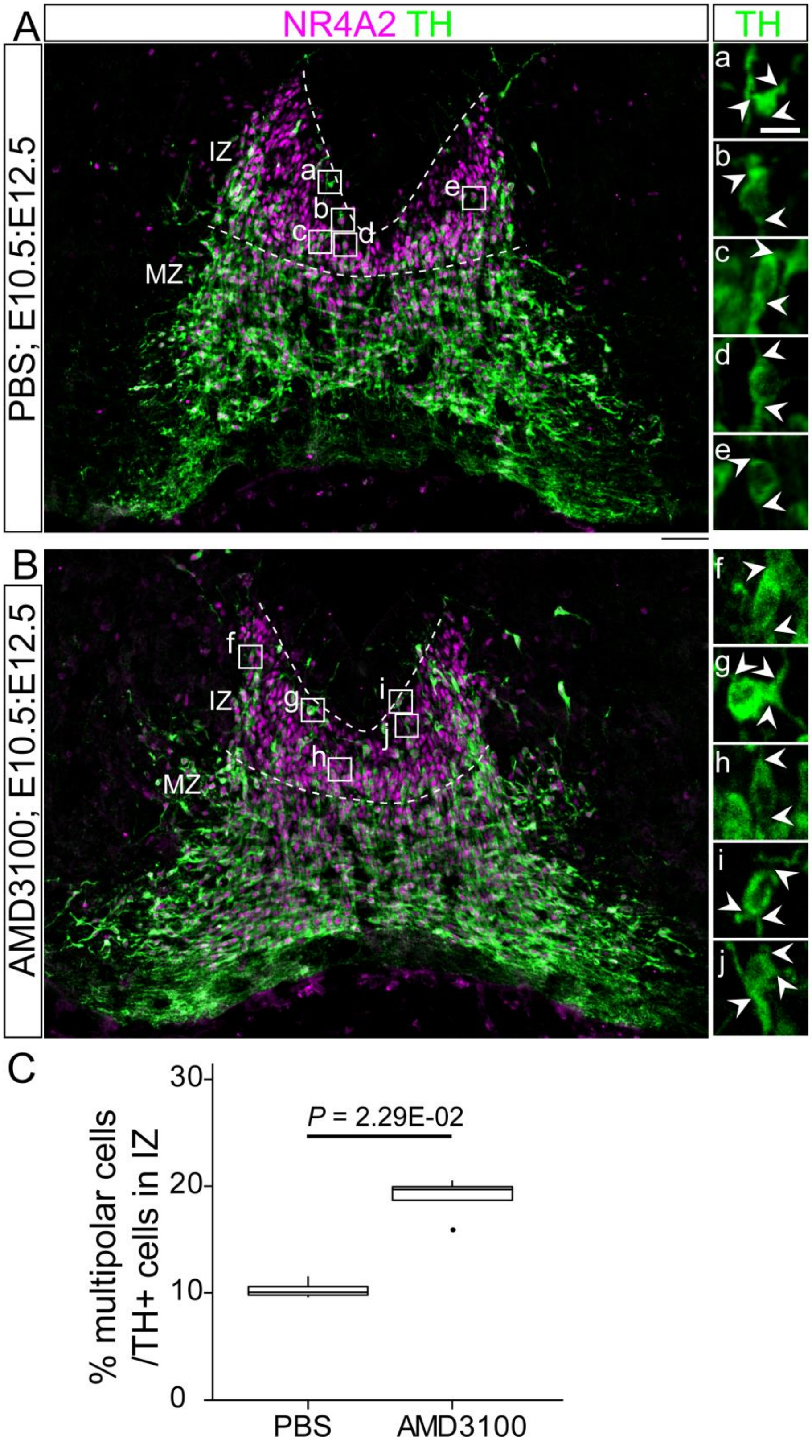

A
NR4A2 TH
TH
PBS; E10.5:E12.5
IZ
MZ
a
b
c
d
e
B
AMD3100; E10.5:E12.5
f
g
h
i
j
C
% multipolar cells /TH+ cells in IZ
P = 2.29E-02
30
20
10
0
PBS
AMD3100

Supplementary Figure 7. Blockage of CXCR4 causes multipolar morphology in dopaminergic neurons. (A, B) Immunofluorescence images show tyrosine hydroxylase (TH; green) and NR4A2 (magenta) in midbrain at E12.5, two days after injection of PBS (A) or AMD3100 (B), a specific CXCR4 antagonist, in mesencephalic ventricle at E10.5. Boxes a-e in (A) and f-j in (B) are magnified in (a-e) and (f-j) respectively to show morphology by TH (green). White arrowheads indicate processes. (C) Percentage of multipolar TH^+ cells out of total TH^+ cells in IZ of PBS (n = 4; median 10.1%, range 9.79%-10.6%) or AMD3100 (n =4; median 19.7, range 18.7%-20.0%) injected midbrain. Scale bars: 50 μm (A, B) and 10 μm (a) for a-e, f-j. Mann Whitney U test was used. IZ: intermediate zone; MZ: marginal zone.

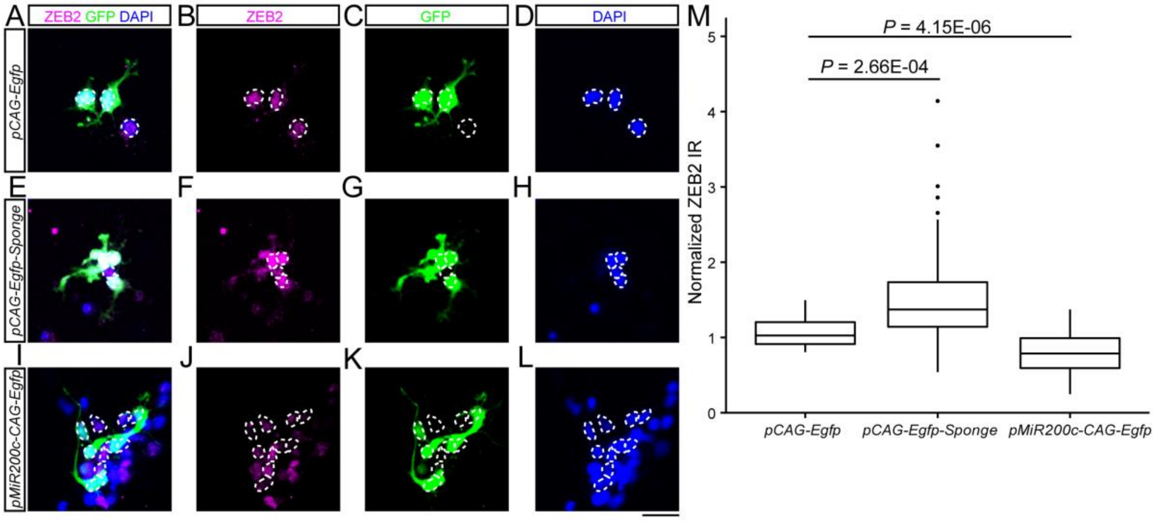

Supplementary Figure. 8. Knock-down of *miR-200c* increases ZEB2 immunoreactivity in primary midbrain cells. (A-L) Immunofluorescence images show ZEB2 (magenta) in primary midbrain cells electroporated with *pCAG-Egfp* (A-D), *pCAG-Egfp-Sponge* (E-H) and *pMiR200c-CAG-Egfp* (I-L) vectors, respectively. Scale bar in A-L: 25 μm. (M) Quantification of ZEB2 immunoreactivity (IR) in GFP^{+} cells electroporated with *pCAG-Egfp* (n =58, N = 3; median 1.02, range 0.911-1.20), *pCAG-Egfp-Sponge* (n = 108, N = 3; median 1.37, range 1.14-1.73) or *pMiR200c-CAG-Egfp* (n = 66, N =3; median 0.788, range 0.589-0.992) plasmids, which is normalized to ZEB2 IR in GFP^{-} cells in the same well to avoid variation between wells. Student t-test was used for statistical analysis.

Supplementary References:

1. van Grunsven, L. A. *et al.* Interaction between Smad-interacting protein-1 and the corepressor C-terminal binding protein is dispensable for transcriptional repression of E-cadherin. *J. Biol. Chem.* **278,** 26135–45 (2003).
2. Jahan, F. Zeb2: A novel regulator of cardiac fibroblast to myofibroblast transition. (The University of Manitoba, 2014).
3. Oztas, E. *et al.* Novel monoclonal antibodies detect Smad-interacting protein 1 (SIP1) in the cytoplasm of human cells from multiple tumor tissue arrays. *Exp. Mol. Pathol.* **89,** 182–9 (2010).
